# Special Issue “Recent Advances in Precision Nanomedicine for Cancer”

**DOI:** 10.3390/molecules25184148

**Published:** 2020-09-10

**Authors:** Chiara Brignole, Fabio Pastorino

**Affiliations:** Laboratory of Experimental Therapies in Oncology, IRCCS Istituto Giannina Gaslini, 16147 Genoa, Italy; chiarabrignole@gaslini.org

**Keywords:** nanomedicine for anti-cancer therapy and imaging, cancer, precision nanomedicine, personalized nanomedicine, silver nanoparticles, variabilin-loaded nanoparticles, intelligent RNA expression device (iRed), RNA interference, cationic liposomes, phage display-based nanotechnology, cancer immunotherapy, functional targets

Nanomedicine, the application of nanotechnology at the level of one billionth of a millimeter to medicine, has inspired great interest in the last twenty years, leading to the commercialization of successful products both from a clinical and an economic point of view. In the clinic, nanomedicine aims to exploit nanotechnology for several biomedical applications, mainly molecular imaging, diagnosis and disease treatment, as well as regenerative medicine and tissue engineering. Recently, precision medicine became an emerging approach for oncology treatment and prevention, which takes into account the individual genetic and phenotypic variability of each patient. In contrast to a one-size-fits-all approach, the precision medicine approach might help to predict more accurately which treatment and prevention strategies will work for specific groups of patients.

In this context, the coupling of nanomedicine and precision medicine for cancer motivated this Special Issue.

In detail, three original articles and two reviews covering some of the most recent advances in precision nanomedicine for cancer are reported. From the beginning, nanomedicine has been frequently associated with the use of nanoparticles in oncology. In this Special Issue, four different nanoparticles are indeed suggested for use in precision nanomedicine for cancer. The three articles deal with the use of nanoparticles encapsulating “anti-tumor” agents of different natures and characteristics. Matysiak-Kucharek M. et al. [1] suggest silver nanoparticles, usually used for their anti-microbial activity, as a novel, personalized anti-cancer drug for triple-negative breast cancer patients, characterized by a lack of expression of estrogen receptor, progesterone receptor and by a non-elevated expression of the human epidermal growth factor receptor (HER2). They report the potential impact of silver nanoparticles on this tumor model, in terms of the cytotoxic effect, based on the disruption of the oxidative balance. However, deeper investigations are needed to exclude any anti-apoptotic changes and pro-inflammatory protein secretion [1]. Lerata M.S. et al. [2] encapsulate a mixture of the natural products (7*E*,12*E*,20*Z*)-variabilin and (7*E*,12*Z*,20*Z*)-variabilin, extracted from the South African marine sponge, Ircinia sp., into solid lipid nanoparticles, with the aim to avoid their general lack of selectivity when administered as a free form. The anti-tumor effects obtained on different solid tumor cell lines demonstrate that stearic acid solid lipid nanoparticles markedly improve variabilin stability and enhance its cytotoxic activity, particularly against a prostate cancer cell line, while sparing non-tumor cell lines. This application might be considered as a novel precision medicine-based approach to overcome some obstacles frequently raised during the development of natural product drugs [2]. Ando H. et al. [3] propose the use of an intelligent RNA expression device (iRed), complexed to cationic liposomes, to augment an in vivo gene silencing effect without eliciting marked innate immune stimulation. The results obtained by the authors demonstrate that iRed-liposome complexes successfully induce efficient gene silencing against pleurally disseminated mesothelioma tumors, while alleviating the innate immune stimulatory effect, paving the way for their widespread utilization as precision nanomedicines for intracelial malignant cancers [3]. Samec N. et al. [4] present an overview of the use of nanoparticles as tools for the diagnosis and treatment of grade IV glioblastoma, an aggressive brain tumor, whose current therapies are ineffective and patient survival rates and prognosis are very poor. The authors suggest that the development of precision nanodevices, specifically targeting cancer cells and endothelial tumor cells through the use of tumor-specific ligands, could improve diagnosis and would allow for enhanced anti-cancer drug solubility, efficient drug transportation through the blood–brain barrier and increased blood circulation half-life. This, in turn, could increase the anti-tumor effects of multiple combined chemotherapy- and immunotherapy-based approaches, which are mandatory due to the high tumor heterogeneity observed in glioblastoma patients [4]. As previously reported, precision approaches also depend on the identification of unique and functional targets on tumor cells and on the tumor vasculature. These targets can be discovered by the use of phage display technology. However, in the second review, Goracci M. et al. [5] also report that phage display-derived peptides can be used not only to decorate “anti-cancer drug”-loaded nanoparticles, but that they can also play a role in cancer immunotherapy, to mimic cancer antigens and/or as small molecule effectors of immune cell functions. Moreover, phages themselves can be suitable carriers for peptides and protein vaccines. Thus, although no commercial phage-derived nanomedicines for cancer immunotherapy are available at present, the authors suggest that such nanotechnological systems have the potential to become, in the future, efficient personalized anti-cancer applications [5].

In conclusion, the research area of precision nanomedicine for cancer is innovative and is growing exponentially, and we believe that some promising applications will reach the clinic soon. We thank all of the authors for their contributions to this Special Issue and the staff members of MDPI for their editorial support.
